# Potential role of geranylgeraniol in managing statin-associated muscle symptoms: a COVID-19 related perspective

**DOI:** 10.3389/fphys.2023.1246589

**Published:** 2023-11-17

**Authors:** Barrie Tan, Kok-Yong Chin

**Affiliations:** ^1^ American River Nutrition, Hadley, MA, United States; ^2^ Department of Pharmacology, Faculty of Medicine, Universiti Kebangsaan Malaysia, Kuala Lumpur, Malaysia

**Keywords:** COVID-19, diterpenoid alcohol, HMG-CoA reductase inhibitors, mevalonate pathway, myopathy, statin

## Abstract

Myopathy is the most common side effect of statins, but it has not been addressed effectively. In anticipation of its wider use as a small molecule to complement the current COVID-19 management, a pharmacological solution to statin-associated muscle symptoms (SAMS) is warranted. Statins act by suppressing the mevalonate pathway, which in turn affects the downstream synthesis of isoprenoids required for normal physiological functions. CoQ10 and geranylgeraniol (GG) syntheses are reduced by statin use. However, CoQ10 supplementation has not been shown to reverse SAMS. GG is an obligatory substrate for CoQ10 synthesis, an endogenous nutrient critical for skeletal muscle protein synthesis. Multiple studies showed GG supplementation is effective in reversing SAMS. This opinion paper proposes employing GG to prevent SAMS in pleiotropic statin use, including usage in the post-COVID-19 pandemic era.

## 1 Introduction

Statins are classic drugs to treat hypercholesterolemia and dyslipidemia (Adhyaru and Jacobson, 2018; Shojaei et al., 2020). Statin’s success in controlling these conditions has gained a statutory body of support from the American Heart Association (AHA), American College of Cardiology, European Society of Cardiology and European Atherosclerosis Society. These societies have promulgated guidelines for atherosclerosis and cardiovascular disease (CVD) risk reduction through lipid management (Feldman et al., 2020). The United States targets LDL of 70–189 mg/dL to reduce CVD risk by 30%, and LDL of >190 mg/dL to reduce CVD risk by 50% (American College of Cardiology/American Heart Association Task Force on Clinical Practice Guidelines, 2019). Europe has a more aggressive “lower-the-better” four-step management targets of “low risk” (LDL <116 mg/dL), “moderate risk” (LDL <100 mg/dL), “high risk” (LDL <70 mg/dL), and “very high risk” (LDL <55 mg/dL) (Mach et al., 2020). In addition, statins have been recommended to patients with diabetes since more than a decade ago in the United States (Eldor and Raz, 2009; American Diabetes Association Professional Practice Committee, 2021). A 10-year-long Veterans Association-Harvard study of >300,000 elderly (>75 years) on statins reported that CVD risk and all-cause mortality were reduced significantly for statin users. Statin consumption can potentially benefit 25 million elderly age >75 years or 45 million elderly age >65 years in the United States. Inflammation reduction by statin in the elderly was the suggested mechanism (Orkaby et al., 2020).

There has been a renewed interest in statins in the pandemic era of Coronavirus disease 2019 (COVID-19). Statins are among the small molecules repurposed to fortify our immune system, along with vaccines, to shield the public against the threat of COVID-19. Retrospective and observational studies have exhaustively demonstrated the benefits of statins among patients with COVID-19 (Table 1). Of note, a CVD/COVID-19 study derived from the AHA/CVD registry of >105,000 patients from >100 hospitals was conducted in 2020. The study reported that Covid-19-related severity for patients previously on statins was reduced by 25% and Covid-19-related death was reduced by 45%. Other researchers found statin use was associated with a reduced risk (by 30%–50%) of developing severe COVID-19 symptoms while speeding up recovery (Daniels et al., 2021; Gupta et al., 2021; Maric et al., 2021). A year later, numerous meta-analyses unequivocally supported that statins reduced the severity and mortality of patients with COVID-19 (Table 2). Nothing in recent history has had statin studies produced at such a rapid pace and in such a narrow time bandwidth. Much larger population groups continued to show statins improved clinical outcomes of patients with COVID-19 [Rivera et al. (2023) with >15,500; Crimi et al. (2023) with >38,800; Wang et al. (2023) with >800,000]. The pandemic threat has accelerated the speed of research. The conclusions and recommendations of the studies presented in Table 1, Table 2 were summarized in Table 3.

**TABLE 1:** Retrospective and observational studies of the effects of statin on COVID-19 outcomes.

References	Study type	Main effects^a^	Findings
Zhang et al. (2020)	Retrospective	↑	In-hospital statin use was associated with 44.7%↓ of all-cause mortality
			Statin benefits may be due to immunomodulation; at 28 d hospital admission C-reactive protein (CRP) (42%↓), interleukin-6 (41%↓), and neutrophil (23%↓) for statin vs. non-statin survivors
Tan et al. (2020)	Retrospective	↑	Statin use was independently associated with 49%↓ of ICU admission
			These dyslipidemia patients have a higher innate immune response (white blood cells ↑8.2% and neutrophils ↑13.8%)
Saeed et al. (2020)	Observational	↑	In-hospital patients with diabetes and COVID-19 on statins had lower CRP (21%↓) and mortality (38.5%↓)
			Patients with diabetes had a 2–3 × greater risk of death with COVID-19; this study was on sicker patients
Chacko et al. (2021)	Retrospective	↑	Antecedent statin use was associated with reduced in-hospital mortality (OR 0.14)
			These were very sick patients on admission (65 years, borderline obese; body mass index (29.5 kg/m^2^), high blood pressure (88%), type 2 diabetes (64%), coronary artery disease (CAD) (34%), end-stage renal disease (ESRD) (14%)
			Worst odds (OR) for in-hospital mortality; Intubation > ESRD > Age > CAD > Male
Umakanthan et al. (2021)	Retrospective	↑	Patients with COVID-19 on statin had lower mortality (44.8%↓) and need of mechanical ventilator (17.4%↓), CRP (17.4%↓) and WBC (8.2%↓)
			Statin use in patients with COVID-19 lowered total cholesterol (6.3%↓) and low-density lipoprotein cholesterol (14%↓), and improved survival and severity
Lee et al. (2021)	Observational	↑	Prior statin use in patients with COVID-19 had 26.3% less severe clinical outcomes and 9.5% shorter hospital stay
El-Solh et al. (2022)	Observational	↓	Statin was not associated with decreased mortality nor ICU admission nor mechanical ventilator use
			A high proportion of statin users were older (66 years) men (91%) known to have higher comorbidities and higher risk of mortality
Shen et al. (2021)	Retrospective	↑	Statin use in patients with COVID-19 had a lower risk of in-hospital mortality (87%↓) than non-users
			This benefit is true for patients with coronary heart disease (CHD) as well as non-CHD patients
Bergqvist et al. (2021)	Observational	↑	Statin treatment had preventive effects on COVID-19 mortality
Kuno et al. (2022)	Observational	↑	In-hospital statin use decreased in-hospital mortality compared to patients who discontinued (34%↓)

^a^ Retrospective and observational studies that improved (↑) and did not improve (↓) in patient severity and death.

**TABLE 2:** Meta-analyses of statin benefits in COVID-19 cases.

References	Main effect^a^	Comments
Gupta et al. (2021)	↑	Antecedent statin use was associated with inpatient mortality
		Those on statins had lower C-reactive protein
Wu et al. (2021)	↑	Statin use showed a reduction in mortality (29%↓) and a need for invasive mechanical ventilators (19%↓)
Yetmar et al. (2021)	↑	Prior statin use was associated with a lower risk of mortality or severity in patients with COVID-19
		Severity is defined by advanced ventilator support, ICU admission, acute respiratory distress syndrome (ARDS)
Zein et al. (2022)	↑	Statin was associated with a lower risk of mortality (28%↓) This association was not affected by age, male gender, diabetes and hypertension
Chow et al. (2021)	↑	Patients given statin *de novo* after COVID-19 diagnosis were at a lower risk for mortality
		Among non-ICU patients, statin users were at a lower risk of mortality relative to non-statin users
Kollias et al. (2021)	↑	Statin therapy was associated with a lower risk of in-hospital mortality (35%↓)
		12 studies (65 years) with comorbidities—hypertension (66%), diabetes (43%), statin use (30%)
Kow and Hasan (2022)	↑	Statin was associated with a lower risk of all-cause mortality and severe illness in patients with COVID-19
Gutierrez-Mariscal et al. (2021)	↑	Statin use was associated with a lower risk of mortality in patients with COVID-19
		Most common comorbidities—hypertension (51%) dyslipidemia (41%), diabetes (33%)
		Only chronic use of statins reduced mortality in patients with COVID-19
Ayeh et al. (2021)	↓	Statin use had no effect on COVID-19-related mortality
		Statin use increased COVID-19-related severity by 18%
		The divergent findings may be due to different demographics and prevalent comorbidities
Choi et al. (2022)	↑	Statin use was associated with a reduction in in-hospital mortality (21%↓)
		Higher-intensity statin use had even more protection than low-to-medium statin use
Vahedian-Azimi et al. (2021)	↑	Antecedent statin use reduced tracheal intubation (27%↓)
		Statin use after hospitalization was associated with 46%↓ mortality

^a^ Meta-analysis studies with statins that improved (↑) and did not improve (↓) in patient severity and death.

**TABLE 3:** Conclusions and recommendations for statin usage to mitigate COVID-19.

Conclusions	
1	Antecedent statin use is associated with a lower risk of severity and mortality
2	Severity decreased and mortality dropped for those on statins
3	Prior chronic use of statin and non-ICU patients fared better
4	Benefits were seen in COVID-19 patients with high cholesterol and inflammation after COVID-19 diagnosis lowered mortality risk
Recommendations	
1	Continue statin in patients with COVID-19 who were already on them
2	Continue statin in CVD and Lipidemia patients without COVID-19
3	Initiate statin therapies among patients at risk of COVID-19
4	Initiate of statin therapies among patients hospitalized with COVID-19
5	Initiate statin therapies among diabetics, especially minorities

The therapeutic function of statin in inhibiting COVID-19 is compelling in two instances—the prior use of statin before COVID-19 infection and the statin treatment *initiation* after COVID-19 infection. In a recent interventional study by Memel et al. (2022), the risk of death was reduced by 43% for patients who *initiated* statin treatment recently. This association held up in patients with statin use (risk of death reduction by 73%) and without statin use (risk of death reduction by 51%) before hospitalization. Benefits were greater with longer exposure to statin before COVID-19, possibly because statin blunts inflammatory response. The authors proposed antecedent statin usage interfered with viral infection via inhibition of cholesterol-rich or lipid-rich cell membranes (Memel et al., 2022).

**FIGURE 1:**
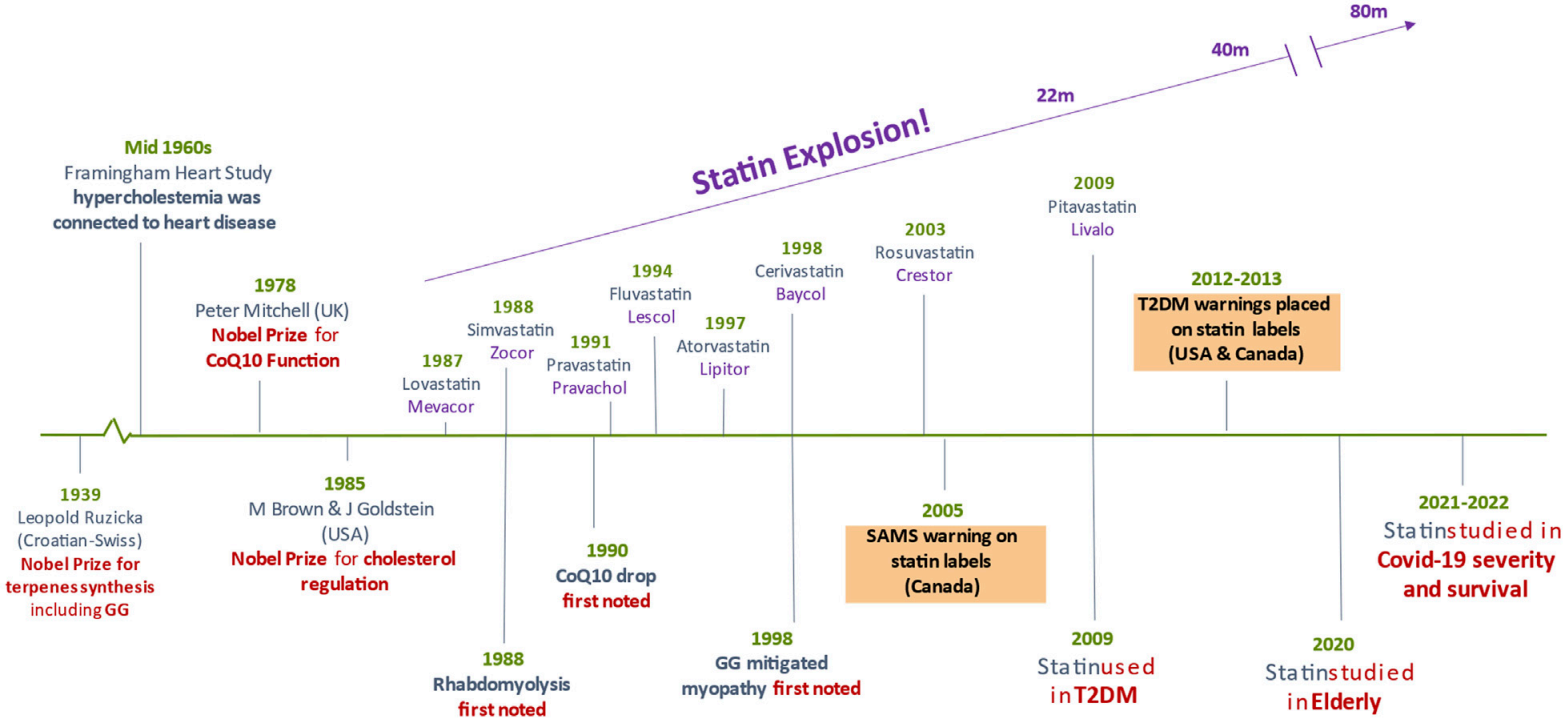
Statin history and landmarks.

With such positive pleiotropic benefits of statins, it is easy to overlook their side effects. The first US commercial statin (lovastatin) was introduced in 1987, and rhabdomyolysis was reported among patients a year later (East et al., 1988; Norman et al., 1988) (Figure 1). This severe form of muscle damage has resurfaced in the current pandemic. Statin users with COVID-19 had more severe and intense muscle pain (≥5%) than non-statin users with COVID-19 (Schetz et al., 2022). In a case study, statin was singled out to cause toxic myopathy and liver damage among other medications (Sabljic and Basic-Juki, 2022). Patients developed proximal muscle weakness (arms and legs) with elevated liver enzymes, whereupon statin cessation normalized enzymes and regression of muscle symptoms. Furthermore, 30%–62% of patients prescribed statins discontinued therapy because of muscle fatigue, weakness and pain (Zaleski et al., 2018).

As yet, statutory bodies have not approved statin use for COVID-19 management. If and when statin is approved for COVID-19, a dramatic rise in statin-associated muscle symptoms (SAMS) will occur. When SAMS are encountered, doctors often advise patients to discontinue statin temporarily until muscle symptoms stop, resume statin at the same or reduced dosage, change to another statin (often lipid-soluble to water-soluble one), use intermittent dosing (taking statin on alternate days) or prescribe non-statin cholesterol-reducers (Altmann et al., 2004; Julius, 2017). These are “bandage” solutions to an unmet problem. Not only is the rate of voluntary discontinuation of statin therapy high, patients often do not consult their doctors on discontinuance. Statin cessation may bring detrimental consequences to patients, including COVID-19 patients as complementary management (Toth et al., 2018; Fedson, 2021).

Given its significance, potential pharmacological agents to address SAMS should be investigated. We propose geranylgeraniol (GG) as a suitable candidate. To explain the rationale for proposing GG, this paper aims to summarise the basic mechanisms of how statins cause SAMS, and the specific role of GG in countering SAMS.

## 2 The molecular mechanism of statins in inducing SAMS

The effectiveness of statins in treating hyperlipidemia is well acknowledged, but the *en route* damage is unappreciated. Statin is an *indiscriminate cholesterol reducer*, by which it reduces endogenous essential isoprenoids besides cholesterol. These isoprenoids are obligatory for the synthesis of other molecules required for normal physiological processes and their decrease would have many downstream ramifications (Verdaguer et al., 2022). Reduction of isoprenoids and the subsequent decimation of skeletal muscle protein and CoQ10 synthesis could be responsible for statin’s side effects (Mollazadeh et al., 2021).

CoQ10 is biosynthesised as an essential nutrient within our body for energy production. The impact of statins on reducing CoQ10 production is well communicated. This CoQ10 reduction is caused by the inhibition of GG synthesis because GG is an intrinsic part of the chemical structure of CoQ10 (Paiva et al., 2005). The health benefits of CoQ10, particularly in cardiovascular health have been documented in many reviews (Martelli et al., 2020; Gutierrez-Mariscal et al., 2021; Rabanal-Ruiz et al., 2021). CoQ10 improves fatigue recovery of elite athletes (Mizuno et al., 2008), and exerts protective effects on the nervous system (Testai et al., 2021) and male fertility (Salvio et al., 2021). Importantly, CoQ10 prevents LDL oxidation (to the atherogenic oxLDL) because ubiquinol (reduced form of CoQ10) is uniquely situated inside LDL particles. All these studies amply underscore the importance of CoQ10 in cardiovascular health and non-cardiovascular health.

Mechanistic studies have variously hypothesised that CoQ10 helps to prevent statin myopathy. However, this CoQ10-SAMS narrative cannot be clinically substantiated as trials and meta-analyses nearly all failed to support the mitigating effects of CoQ10 on SAMS (Table 4). Supplemental CoQ10 that drives serum CoQ10 levels up after being lowered by statin *implies* CoQ10 could mitigate SAMS. A specific trial was designed to test this hypothesis. In this study, statin users with confirmed myalgia were supplemented with 600 mg CoQ10 per day for 8 weeks. Their serum CoQ10 rose from 1.3 μg/mL to 5.2 μg/mL, an impressive 400% increase above baseline (Taylor et al., 2015; Zaleski et al., 2018). There is no CoQ10 “bioavailability” issue in this trial. However, CoQ10 did not improve skeletal muscle symptoms or performance in patients with SAMS. Thus, statin-associated myopathy could not be effectively managed by CoQ10 supplementation.

**TABLE 4:** The effects of CoQ10 supplementation on statin-associated muscle symptoms (SAMS).

References	Study	Main effect^b^	Comments on CoQ10 in patients on statins
Banach et al. (2015a)	Meta-analysis	↓	CoQ10 did not cause any difference in muscle pain
Banach et al. (2015b)			No change in phosphocreatine kinase (PCK)
Taylor et al. (2015)^a^	Clinical trial	↓	Patients with confirmed myalgia
Zaleski et al. (2018)^a^			CoQ10 (600 mg/d for 8 weeks); serum CoQ10 increased 400% (1.3 μg/mL to 5.2 μg/mL)
			CoQ10 did not improve skeletal muscle symptoms nor performance in patients with confirmed SAMS
			PCK fluctuated too widely to be useful
Qu et al. (2018)	Meta-Analysis	↑	Twelve studies were included in the analysis
			CoQ10 ameliorated SAMS
			Supplementation as a complementary approach
			No reduction of PCK
Kennedy et al. (2020)	Meta-Analysis	↓	Seven studies were included in the analysis
			CoQ10 did not improve myalgia symptomsCoQ10 did not improve adherence to statin therapy; patients discontinued medication
Durhuus et al. (2020)	Clinical trial	↓	Statin patients with and without myalgia on CoQ10 (8 weeks with 400 mg/d)
			CoQ10 had no impact on mitochondrial functions nor was it bioavailable (platelets and PMBC)
			ROS increased in platelets of statin takers with SAMS (250%↑) and without SAMS (100%↑)
Wei et al. (2022)	Meta-Analysis	↓	Eight studies were included in the analysis; 4 evaluated CoQ10 on muscle pain
			CoQ10 did not improve statin-induced myopathy
			PCK increased NS
Chen et al. (2022)	Retrospective	↓	12.5% of statin takers (*n* = 511) also supplemented CoQ10 (*n* = 64)
			The frequency of SAMS resolution was similar between groups
			CoQ10 did not improve their muscle symptoms

^a^ This study is considered the most rigorous in defining SAMS, to study with CoQ10 supplementation.

## 3 Role of GG in managing SAMS

**FIGURE 2:**
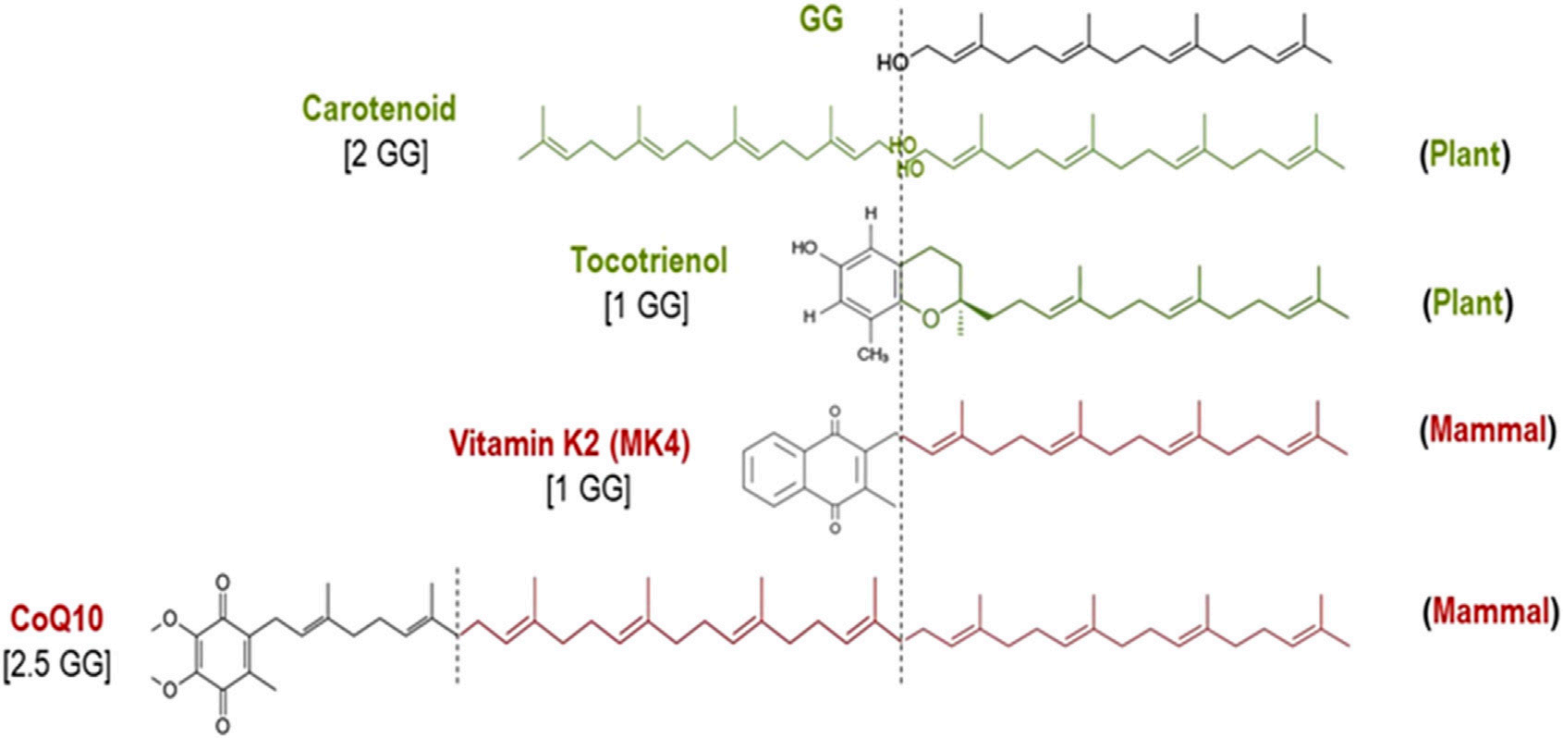
GG as a building block of biomolecules in plants (green) and mammals (red).

**FIGURE 3:**
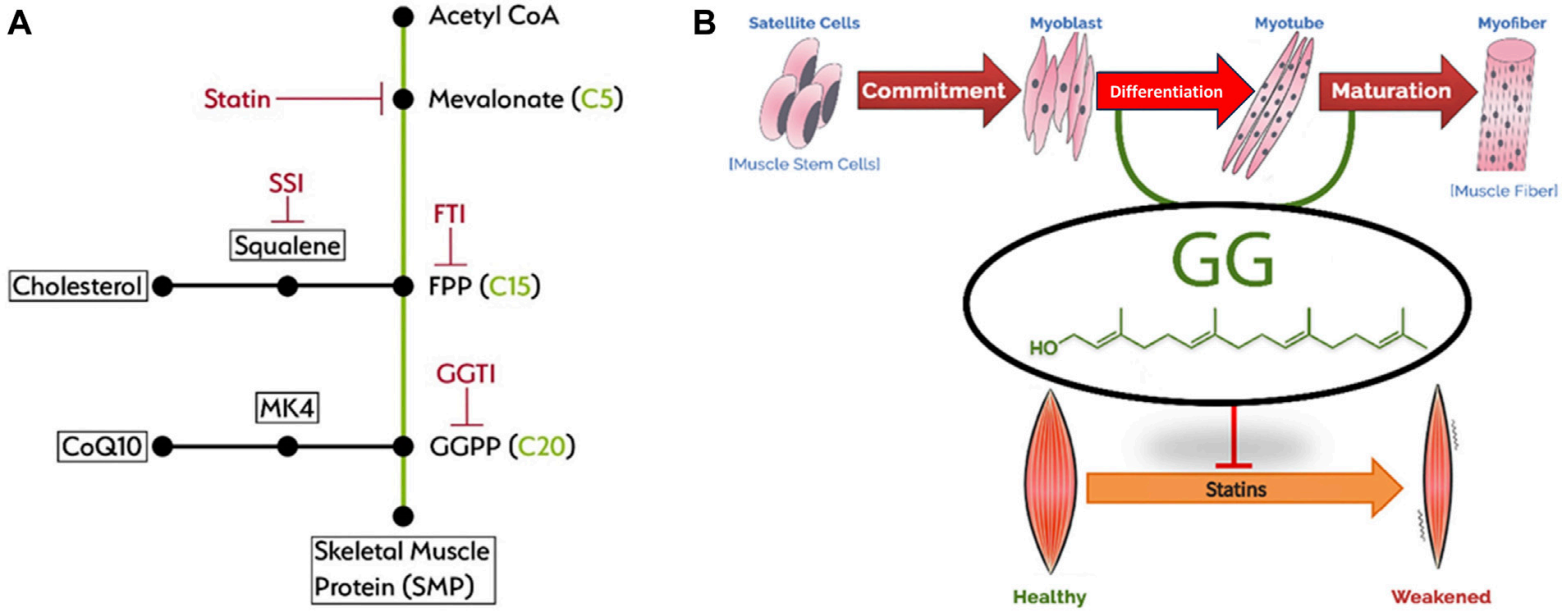
Mevalonic acid pathway **(A)** and statin myotoxicity **(B)**.

The biochemical and biological pathway of GG to mitigate muscle cell damage due to statins was elegantly studied >25 years ago (Raiteri et al., 1997) and confirmed >15 years ago (Johnson et al., 2004). Statin causes apoptosis of muscle cells with a concomitant loss of GG. Cholesterol, CoQ10 and menaquinone-4 (MK4) were all decreased by statin. GG is the “building block” isoprenoid for the synthesis of CoQ10 and MK4 in animals (Nakagawa et al., 2010; Hirota et al., 2015) and also for the synthesis of ubiquitous phytonutrients in plants (Figure 2). The adverse effects of statins on muscle are mainly through inhibition of protein geranylgeranylation, but not by ubiquinone suppression (Johnson et al., 2004). GG add-back studies showed increased CoQ10 production without blocking statin’s inhibition of cholesterol synthesis (Campia et al., 2009). Both farnesol (F) and geranylgeraniol (GG) are isoprenoids central to the mevalonic acid pathway. Cholesterol biosynthesis is derived from F. Statin’s inhibition of this pathway is much upstream at the C5 mevalonate site for which F (at C15) and GG (at C20) both undergo downstream obligate inhibition (Figure 3). This mevalonate-isoprenoid pathway is rendered more plausible because CoQ10, ezetimide, and statin—all related to cholesterol synthesis—were summarily associated with reduced COVID-19 hospitalization risk (Israel et al., 2021; Sumbalova et al., 2022). Since a higher CoQ10 level was associated with reduced hospitalization, the authors suggested that isoprenoid GG reduction is responsible for hospitalisation. GGPP (activated form of GG) is required for the synthesis of CoQ10. Interestingly, Health Canada has required myopathy warnings on statin labels for more than 15 years (Health Canada, 2005) (Figure 2).

One novel mechanism for which statin inhibits COVID-19 is via the mevalonate-isoprenoid reduction of FPP/cholesterol. There is a remarkable similarity to the pleiotropic statin effects on the cardiovascular system (Oesterle et al., 2017). GG is uniquely required for the synthesis of skeletal muscle protein. GG increases the differentiation of muscle cells and suppresses the expression of skeletal muscle atrophy-related ubiquitin ligases (Matsubara et al., 2018) (Figure 3). Statins obligatorily inhibit this GG isoprenoid and prevent the prenylation of skeletal muscle protein, thus myopathy occurs. GG is a non-terminal nutrient, the “missing piece” in skeletal muscle protein. Several *in vivo* models have showcased the potential of GG in improving muscle health. First, GG prevents statin-induced muscle fatigue by increasing force production and muscle performance (Irwin et al., 2020). Second, GG prevents denervation-induced reduction in muscle fibres (Miyawaki et al., 2020). Third, GG prevents degenerations of soleus muscle in diabetic animals by improving mitochondrial quality and autophagy (Jiwan et al., 2022). In a related *in vitro* study, GG also rescues mitochondrial respiration in monocytic cells suppressed by statins, likely due to GG being used for CoQ10 synthesis (Campia et al., 2009). In addition, GG interacts with the prenyltransferase UbiA prenyltransferase domain-containing protein l, which converts menadione to menaquinone (MK4) (Schumacher et al., 2015). MK4 is important for the maintenance of a healthy musculoskeletal system (Azuma and Inoue, 2019). However, there is a lack of direct evidence suggesting GG promotes MK4 synthesis. Overall, the accumulated evidence suggests the beneficial effects of GG in protecting muscle health.

The extreme case of statin-induced muscle problem, rhabdomyolysis, was noted in 1988, merely a year after the first statin was introduced in the United States. The decimation of endogenous GG was identified as the responsible cause (Raiteri et al., 1997; Johnson et al., 2004). Yet, it would take another 25 years to readdress GG as being responsible for skeletal muscle protein and its deterioration by statins. Arguably, the most prevalent side effect of statins is muscle problems, and this damage can be studied through the development of muscle atrophy. Muscle atrophy is the loss of skeletal muscle mass, decrease in force production and increase in fatigue. Atrophy can happen with neuromuscular and musculoskeletal diseases, aging (loss of testosterone), immobility/sarcopenia (use-it-or-lose-it), and exercise-induced muscle injury.

Loss of CoQ10 causes fatigue, and myalgia, but the loss of GG goes beyond energy deficits to muscle damage and atrophy. Supplemental CoQ10 compensates for CoQ10 loss caused by statins. However, supplemental GG reverses SAMS and regenerates CoQ10 (Campia et al., 2009; Sanvee et al., 2021a; Sanvee et al., 2021b). These functions of GG are summarized in Table 5. Put together, GG is the critical isoprenoid responsible for skeletal muscle protein synthesis. GG and F are isoprenoids used in the cell membranes to prenylate proteins. Hanai et al. (2007) first noted that atrogin-1 is the critical mediator of muscle damage (muscle fibre breakdown) caused by statin. Later, the same group discovered that GG—but not F—specifically rescued muscle damage from statins (Cao et al., 2009). Statin reduced myofibers by 60% and only GG completely reversed it. Furthermore, GG reduced statin-induced atrogin-1 by 65%, a mechanism responsible for muscle atrophy. The authors suggested that geranylgeranylation was required for the synthesis of the long CoQ10 sidechain. CoQ10 plays an integral part in the mitochondrial respiratory complex and statin suppresses mitochondrial function (Apostolopoulou et al., 2015). The decrease in the latter was responsible for muscle atrophy. Additionally, MK4 and GG improve mitochondrial functions by increasing mitochondrial membrane potential, mitochondrial respiratory capacity, ATP production, and spare respiratory capacity. The authors remarked that MK4 and GG both contribute to the inhibition of insulin resistance in skeletal muscles (Su et al., 2021; Wang et al., 2022). This statin-damage work has also been substantiated in animal studies (Goodman et al., 2015; Irwin et al., 2018). Oral administration of GG rescues muscle atrophy through atrogin-1 suppression (Miyawaki et al., 2020) and reverses skeletal muscle atrophy that includes oral frailty such as oral conversation and food mastication (Shirakawa et al., 2021). In muscles, statin increased atrogin-1 by about 100%–150%, followed by a consequent reduction of 30%–35% muscle force production for which GG completely abrogated. The translated dose for a 70 kg person is approximately 170 mg/d of GG. GG supplementation increased force production in muscle (Irwin et al., 2020). In an *ex vivo* human study, statin caused the induction of atrogin-1 by ≥ 400%, an indication of muscle damage and muscle atrophy (Gouni-Berthold et al., 2013). These studies showed GG reduced atrogin-1 to reverse muscle atrophy.

**TABLE 5:** Functions of geranylgeraniol (GG) in muscles and muscle cells.

Studies	Effects of GG
Sanvee et al. (2021a)	GG prevented toxicity (caused by statin) in myoblasts (by 400%) and myotubes (by 100%)
	GG improved energy production (decreased by statin) in myoblasts (by 50%) and myotubes (by 30%)
	Statin inhibited cellular respiration and increased superoxide production; GG completely prevented statin-associated membrane toxicity
Sanvee et al. (2021b)	Isoprenoid replenishment (especially by GG) prevented the cytotoxicity associated with statin
	GG prevented cytotoxicity presented by toxic effects of statin in skeletal muscle cells
	GG restored phosphorylation and prevented ATP depletion completely of muscle cells caused by statin
	Statin impaired mitochondrial respiration and caused reactive oxygen species (ROS) production; GG prevented ROS completely, likely interfering with ROS generation
Ownby and Hohl (2002)	Statin led to visible muscle and muscle cell damage; GG reversed and restored myotube and myofiber morphology, inhibition of muscle atrophy via atrogin-1 reduction
Cao et al. (2009)	
Marcuzzi et al. (2016)	
Irwin et al. (2020)	Statin reduced force production of hind legs; GG completely abrogated this effect
Miyawaki et al. (2020)	GG increased shinbone muscle force, reversed skeletal muscle fatigue and improved cardiac muscle contraction/relaxation

## 4 Conclusion

The existing pleiotropic reach of statin is already large. The potential utility of statin to mitigate COVID-19 makes the extended reach of this drug enormous. Assuming a generous two-third overlap of all the possible 240 million statin takers, it is expected that by 2030, 80 million Americans may be on statins (Table 6). Statins stand to topple the most-used drug paracetamol or acetaminophen (CHPA, 2020). With the off-label prescribed statins to the elderly and use in the diabetic population, SAMS is expected to increase. Furthermore, if statins are approved for use in COVID-19 patients, and for patients with post-acute sequelae of COVID-19 (PASC) or simply long-haul COVID-19, muscle problems are staged to multiply. GG addresses this unmet need. CoQ10 is, at best, *a partial* statin solution, whereas GG may be a better solution to address SAMS. CoQ10 and GG combination may be used as a “statin companion” to satisfy the unmet need generated by statin’s usage. The current NIH guidelines recommend continued use of statins during COVID-19 but do not recommend initiation pending completion of randomized controlled trials. The perspectives put forward in this paper will have to be validated in future studies.

**TABLE 6:** Projected number of statin users for its pleiotropic benefits by 2030.

Condition/Situation (usage)	Reach in United States	
Lipidemia (Cholesterol ↑)	40 m	Recommended by American Heart Association
Diabetes (Sugar ↑) Eldor and Raz, (2009)	35 m	Recommended by American Diabetes Association
Elderly (75+) Orkaby et al. (2020)	25 m (45 m if the age is 65+ years)	No recommendation by American Gerontology Society
CVD with COVID-19 Daniels et al. (2021)	120 m (CHD, HF, Stroke, HBP)	Study supported by American Heart Association; no recommendation made

## Data Availability

The original contributions presented in the study are included in the article/Supplementary Material, further inquiries can be directed to the corresponding author.
